# Hamstring Strength and Architectural Properties Are Associated with Running Biomechanics

**DOI:** 10.3390/muscles4040044

**Published:** 2025-10-13

**Authors:** Nicholas Ripley, Christopher Bramah, Paul Comfort, John McMahon

**Affiliations:** 1 School of Health and Society, University of Salford, Salford M5 4WT, UK; c.a.bramah@salford.ac.uk (C.B.); p.comfort@salford.ac.uk (P.C.); john.mcmahon@hawkindynamics.com (J.M.); 2 Manchester Institute of Health and Performance, Manchester M11 3BS, UK; 3 School of Medical and Health Sciences, Edith Cowan University, Joondalup 6027, Australia; 4 Hawkin Dynamics, Westbrook, ME 04092, USA

**Keywords:** eccentric strength, fascicle length, bicep femoris, spatiotemporal, hamstring strain injuries

## Abstract

Applied muscular strain and hamstring strain capacity have a joint interaction on hamstring strain injury (HSI) with modifiable risk factors frequently assessed. However, to date there is limited observations on the interaction between these factors. The purpose of the present study was to observe if spatiotemporal characteristics, running kinematics and muscle activation were related to modifiable risk factors of HSI. Twenty-two competitive team sport athletes (24.7 ± 4.3 years, 1.82 ± 0.07 m, 84.9 ± 8.5 kg) participated whereby the Bicep femoris long head (BF_LH_) fascicle length assessed via ultrasound and isokinetic eccentric hamstring strength was assessed. With running assessment performed at 18 km/h, capturing running kinematics and muscle activation. Multiple linear regressions were used to examine the relationship of running kinematics and muscle activation on the modifiable risk factors of HSI on. The overall model (F_2,19_) was statistically significant for both relative eccentric hamstring strength (F = 23.58, *p* < 0.001) and BF_LH_ fascicle length (F = 18.87, *p* < 0.001) highlighting spatiotemporal characteristics, running kinematics and hamstring activation were found to be significantly related to the modifiable risk factors. There is a complex interrelationship between running mechanics and hamstring muscle properties, with the potential of either cause or consequence association.

## 1. Introduction

The incidence of hamstring strain injuries (HSIs) within sport frequently result from performing one of two high risk actions, kicking or high-speed locomotion [1], with inciting events occurring in phases of acceleration and deceleration [2]. The elevated risk of a HSI occurrence during these high velocity actions is due to the requirement for the hamstrings to produce high forces (i.e., up to 10.5 N/kg for the bicep femoris long head [BF_LH_] during the terminal swing phase) [3], to resist rapid knee extension [1,4,5,6]. An eccentric muscle action is proposed to occur during the terminal swing phase of sprinting based on three-dimensional (3D) modelling [7,8,9,10,11,12,13], although this is contested within the literature with Van Hooren and Bosch [14] postulating that an isometric muscle action occurs during the terminal swing phase based on animal models. Recent research has added further ambiguity to this area; Yoon et al. [15] observed that the BF_LH_ fascicle actively shortens during the late swing phase; however, they also observed asynchronous behaviour of the muscle-tendon unit (MTU). The asynchronous action observed with the MTU could suggest that length changes may occur primarily in the tendon rather than the muscle at increasing sprinting velocities [15]. This could support the notion from Van Hooren and Bosch [14] whereby high forces generated during an eccentric muscle action are the resultant cause of a HSI event [14,16,17]. However, it is important to acknowledge that these findings may partially reflect methodological limitations associated with ultrasound imaging and modelling of dynamic movements at high velocities.

Despite the ambiguity on what muscle action is occurring during the terminal swing phase, eccentric hamstring strength is strongest modifiable predictor of HSI risk and has been shown to reduce the risk of HSIs [18,19,20]. The results of hamstring strength training interventions have indicated that the hamstring muscles adapt rapidly to the stimulus applied, specifically with the inclusion of an eccentric training stimulus, there is a rapid increase in both eccentric hamstring strength and BF_LH_ fascicle length [21]. Both of these adaptive responses to eccentric hamstring focused strength training have not only demonstrated reductions in HSI occurrence [20,22,23], but also subsequent increases in performance of athletic tasks such as sprinting and jumping [24,25,26,27]. Recently, absolute measures of eccentric hamstring strength, assessed using an isokinetic dynamometer, and BF_LH_ fascicle length were shown to have no meaningful association, contrastingly when measures were taken in relative to body mass (relative eccentric hamstring strength) and a measure of relative BF_LH_ fascicle length (i.e., relative to the anatomical distance between attachments sites [ischial tuberosity and lateral tibial epicondyle]), this markedly increased with moderate to nearly perfect relationships observed [28].

Individuals with impaired hamstring muscle function, through either a history of HSI occurrence or acute fatigue, have demonstrated alterations in running kinematics, kicking mechanics, muscle activation patterns, and lengthening muscle tissue mechanics [5,29,30,31,32,33,34]. Researchers demonstrated that a previous HSI significantly reduced horizontal force production during high speed running (~80% maximum velocity) [33], potentially resulting in a decrease in peak hip flexion and the peak knee extensor moment that occurred during the late swing phase of running, although these differences were not assessed [33]. Under fatigued conditions, where it would be expected that the ability for the hamstrings to produce force would be impaired, healthy soccer players displayed significant differences in swing phase kinematics in comparison to non-fatigued conditions [34]. Contrastingly, Silder et al. [32] demonstrated no significant differences in mechanics or muscle activation between previously and non-previously injured limbs when running at 60-, 80-, 90-, and 100% of maximum sprinting speed. However, the impact of an HSI event on hamstring and athletic performance have been made retrospectively [5,29,30,31,32,33], where the changes in kinetics, kinematics, and muscle activation could be a result of motor adaptation following injury in order to optimise and/or to protect the system from further injury [35]. In a recent review on the current evidence for hamstring kinematics [36], the authors highlighted that as the primary mechanism of HSI is strain and that several kinematic parameters directly influence strain running kinematics could represent a modifiable risk factor for future injury [36]. However, as there is not a single driver to kinematic changes in muscular strain suggesting it is potentially an interaction between several kinematic features, such as lumbo-pelvic control, anterior pelvic tilt, forward trunk lean, trunk lateral flexion, maximal hip flexion, and likely the under pinning kinetic features, such as force production, which undoubtedly links to sprinting kinematic characteristics [36,37,38]. Currently many of these findings are based on retrospective studies and therefore further investigation is warranted.

Applied muscular strain (i.e., running kinematics) and the hamstring strain capacity, specifically the BF_LH_ fascicle lengths and eccentric hamstring strength, have a joint interaction on HSI [36]. Bramah et al. [36] highlighted that lumbo-pelvic control as a parameter has the strongest level of evidence to support the risk of HSI; however, it was noted that no single biomechanical parameter has been identified as a driver for applied hamstring strain or HSI risk. Therefore, a combination of parameters including force production characteristics or strain capacity should be considered and monitored, highlighting the need for a combined observation of key features associated with applied strain and strain capacity. Quantitative evaluation of these factors requires methods capable of capturing both external motion and internal tissue behaviour. Three-dimensional (3D) motion capture is considered the gold standard of kinematic assessments, allowing for the quantification of joint angles, angular velocities, and segment coordination during high-speed running [36,39]. In contrast, hamstring strain capacity can be evaluated through a combination of architectural assessments via ultrasound imaging to determine fascicle length and pennation angle [40,41,42], and force-based measurements, with isokinetic dynamometry providing a reliable method of assessing eccentric strength [43]. However, to date there are limited observations on the interaction between these factors. Recently, meaningful relationships have been identified between eccentric hamstring strength and late swing phase mechanics at the knee [44], with the potential that running kinematics or the task specific mechanical demands may result in physical and architectural adaptations [45,46]. Moreover, on completion of a four-week Nordic hamstring exercise training programme, improvements in the late swing phase knee mechanics were also observed [47]. The aim of the present study was to observe if spatiotemporal characteristics, running kinematics and muscle activation was related to relative eccentric hamstring strength and BF_LH_ fascicle length. It was hypothesised that running kinematics would have a relationship with the modifiable risk factors of hamstring strain injuries.

## 2. Materials and Methods

### 2.1. Experimental Setup

An observational experimental design was performed to determine the effect of running kinematics and muscle activation on the modifiable risk factors of HSIs (i.e., BF_LH_ fascicle length and eccentric hamstring strength). A priori sample size estimation based on the differences observed by Sever et al. [48] where the smallest correlation between hip strength and running kinematics (r = 0.68, f^2^ = 0.86), a priori alpha level of 0.05, minimum statistical power of 80% resulted in a required sample size of 15.

Participants were observed on two separate occasions within a one-week period, each interspersed by ≥48 h at the same time of day. Participants’ modifiable risk factors were assessed during the first testing occasion whereby BF_LH_ fascicle length and isokinetic eccentric strength measurements for hamstrings [28]. On the second testing bout, participants performed a submaximal treadmill assessment running at 18 km/h with both 3D and EMG measurements taken [49].

### 2.2. Participants

Twenty-two male competitive team sport athletes (Tier 1–3 according to McKay et al. [50]) who incorporated high-speed running (24.7 ± 4.3 years, 1.82 ± 0.07 m, 84.9 ± 8.5 kg) participated within the present study. None of the participants reported that they had previously performed any structured sprint training, with exposure to technical elements being described during sport-based warm-ups. The study was approved by the institutional ethics committee (HSR1718–040) providing written consent. The study also conformed to the principles of the Declaration of Helsinki (2013).

### 2.3. Procedures

#### 2.3.1. Muscle Architecture

BF_LH_ muscle architecture images were collected in a prone position with the hip in neutral and the knee fully extended. All images were collected at the halfway point between the ischial tuberosity and the knee joint fold along the longitudinal axis of the muscle belly utilising a two-dimensional, B-mode ultrasound (MyLab 70 xVision, Esaote, Genoa, Italy) with a 7.5 MHz, 10 cm linear array probe with a depth resolution of 67 mm. A layer of conductive gel was placed across the probe; the probe was then placed on the skin over the scanning site perpendicular to the skin. During collection of the ultrasound images, minimal pressure was applied to the skin. The assessor manipulated the orientation of the probe slightly if the superficial and intermediate aponeuroses were not parallel. These methods are consistent to those used previously by the same authors [40]. Imaging of both limbs took between 8 and 12 min per participant.

Sonograms were analysed offline with Image J version 1.52 software (National Institute of Health, Bethesda, MD, USA). Images were calibrated to the known field of view, then a fascicle of interest was identified. Muscle thickness, pennation angle, observed fascicle length, and distance between fascicle end point and super fascial aponeurosis were measured 3 times within each image, to enable complete fascicle length estimation using a previously established reliable linear equation [40]. Bicep femoris estimation is given by the following equation: fascicle length = L + (h ÷ sin(β)).

Where L is the observable fascicle length, h is the perpendicular distance between the superficial aponeurosis and the fascicles visible end point, and β is the angle between the fascicle and the superficial aponeurosis.

#### 2.3.2. Isokinetic Eccentric Strength

Participants performed a standardised warm-up following the collection of ultrasound images, including 5 min of submaximal cycling, followed by 2 sets of 5 repetitions of body weight squats, forward lunges, and leg swings. Isokinetic strength of the knee flexors was assessed using an isokinetic dynamometer (125 AP, KinCom, TN, USA) sampling at 120 Hz. Participants were seated and secured to avoid secondary joint movement, with the hip flexed to 90°. The range of motion (ROM) of the knee was determined as 0–90° (i.e., full extension to 90° of flexion), and the limb length and limb weight for each subject were recorded, with limb weight being measured at rest at 0°, for gravitational correction during data analysis [43].

Eccentric knee flexion was at a single standardised angular velocity (60°·s^−1^), as this angular velocity to detect future HSI risk [19]. Subjects performed 5 submaximal incremental repetitions, which were used for familiarisation purposes. Three maximal eccentric knee flexion efforts were performed, and a 60 s rest period was provided between each repetition. Participants were instructed to pull the dynamometer head as “hard and fast as possible”, with strong verbal encouragement provided during the task.

Raw torque/angle data was analysed using a custom designed Excel spreadsheet (Microsoft, Redmond, WA, USA). Phases of acceleration and deceleration were initially deleted from the analysis using a tolerance of ±1°·s^−1^, with the included isokinetic range gravity corrected.

#### 2.3.3. Three-Dimension and Task Electromyography

Following a standardised warm up, which is crucial for the collection of EMG data [51]. All 3D motion data was collected over a 15 s duration using infrared cameras (250 Hz) operating through Qualisys Track Manager (QTM) (Oqus 7+, Qualisys AB, Partille, Sweden) on a single treadmill (T9450 HRT Vision Fitness, Cottage Grove, WI, USA) for all running trials which was situated within the 3D motion capture area. Retro-reflective markers and cluster sets of four markers were placed onto the body landmarks to define the pelvis, thigh, shank, and foot segments.

Surface EMG data captured at 1500 Hz through QTM of BF_LH_ and medial hamstrings (MH)(semitendinosus [ST] and semimembranosus) was measured for all trials. Prior to electrode placement, the participants’ skin was prepared using a standardised process of shaving (with a disposable safe razor), rubbing with a preparation gel and cleaning with an alcohol-based solution. Skin preparation was performed to minimise resistance (i.e., to reduce inter-electrode resistance to values below 5 kΩ [52]. Ag-AgCl electrodes with wireless EMG sensors and a reference pad (Noraxon U.S.A. Inc., Scottsdale, AZ, USA) were placed on to the surface of the skin of both limbs attached in orientation with the muscle fibres. Electrodes were placed at the mid-point of the BF_LH_ and the medial hamstrings. Correct electrode placement was confirmed prior to commencing data collection with manual muscle testing (i.e., by asking the participants to voluntarily contract the hamstrings against manual resistance) and minimal cross-talk will be visually and physically checked via internal and external rotation of the leg with a 90° knee angle, as per Timmins et al. [53].

A maximal treadmill sprint assessment was performed. This method was chosen to allow for comparisons of EMG attained at the sub-maximal running speed to be expressed as a percentage of maximal velocity. Following the sub-maximal treadmill trial, a 5 min rest period was provided. Participants performed a maximal treadmill sprint assessment to normalise task EMG data. This method was chosen to allow for comparisons of EMG attained at the sub-maximal running speed to be expressed as a percentage of maximal velocity. Following the sub-maximal treadmill trial, a 5 min rest period was provided. Participants ran at increasing velocities where they were required to maintain a set running velocity for 10 s with 180 s recovery between each trial. Commencing at 18 km·h^−1^, with subsequent increases in velocity of 1.5 km·h^−1^ for each running interval, consistent with Numella et al. [54]. They continued this until they could not maintain the pace for the given duration or a rating of perceived exertion (RPE) of >9 was given, when using a scale of 1–10. This was performed on a high-speed treadmill (Woodway Ergo ELG55, Weil am Rhein, Germany), which was in a fixed position outside the 3D motion capture area.

A lower extremity six degrees of freedom kinematic model was created for each participant including the pelvis, thighs, shanks, and feet using Visual 3D (V3D) (C-motion, version 3.90.21, Gothenburg, Sweden). The model utilised a CODA pelvis orientation to define the location of the hip joint centre [55]. The knee and ankle joint centres were defined as the mid-point of the line between lateral and medial markers. Hip and knee angles during the gait and sagittal plane knee and hip joint angles were determined based on the 3D coordinates of one segment to another. Prior to exporting the first derivative kinematic angular data, an 8 Hz low pass filter was applied to the data to attenuate noise [56].

To identify three full strides for the left and right limb, gait characteristics for the left and right foot, take off (TO) and touch down (TD) events were identified. TO was identified as the moment the fifth metatarsal ascended (Z) to a height greater than this minimum threshold (0.22 m) for a minimum of 25 frames (0.1 s). Alternatively, TD was identified as the moment the fifth metatarsal reached 0.22 m for a minimum of eight frames (0.032 s). Following the identification of gait events, data was stride normalised from TD to subsequent TD, with contact time being defined as TD to TO. A description of kinematic running metrics identified from 3D motion data is presented in Table 1.

The raw EMG signals were high- and low-pass filtered (10 and 1000 Hz) to remove unwanted artefacts (noise artefacts (e.g., movement of the cables) and the identification of cardiac signal amplitude). Within a custom Excel spreadsheet, a root mean square filter was applied to the EMG data across a moving average window of 25 ms. Peak EMG amplitudes of the BF_LH_ and medial hamstrings were identified across the normalised stride, in addition to a ratio of the BF_LH_ to MH.

### 2.4. Statistical Analyses

Statistical analyses were performed using JASP software (JASP Team, 0.19.3, [Computer software]). Statistical significance was set at *p* < 0.05 for all tests. Data is presented as mean ± standard deviation (SD). Absolute and relative between-trial and between-stride reliability was assessed by coefficient of variation (CV) and a two-way random effects model intraclass correlation coefficient (ICC), with 95% confidence intervals (CI). Minimum acceptable reliability was confirmed using an CV < 10%. The ICC values were interpreted based on the lower bound CI as (<0.50) poor, (0.50–0.74) moderate, (0.75–0.90) good and (>0.90) excellent [57].

A series of multiple linear regression analyses were conducted, for each kinematic and EMG variable, a distinct regression model was built. The modifiable risk factors (i.e., relative eccentric hamstring strength [N/kg] and bicep femoris fascicle length) set as the dependent variables with the spatiotemporal, kinematic, and EMG data were entered simultaneously as independent predictor variables (using the “Enter” method). The assumptions of multiple linear regression were assessed for each model. Linearity was checked by examining residual plots, and homoscedasticity was assessed by visually inspecting the scatterplot of standardised residuals versus predicted values. The normality of residuals was evaluated using Q-Q plots and histograms of the standardised residuals. Multicollinearity among predictor variables was assessed using Variance Inflation Factor (VIF) and Tolerance values; VIF values below 5 were considered acceptable. The Durbin–Watson statistic was used to check for the independence of residuals.

For each regression model, the overall model fit was evaluated using the F-statistic and its corresponding *p*-value, along with the adjusted R^2^ to indicate the proportion of variance in the dependent variable explained by the predictors. The unique contribution of each independent variable was assessed by examining its standardised (β) regression coefficients, along with their 95% confidence intervals, t-statistic, and associated *p*-values. A statistical significance level of α = 0.05 was set for all analyses.

## 3. Results

All data was determined as being normally distributed (*p* > 0.05). Mean ± SD, CV% and ICC values are presented in Table 2.

A multiple linear regression was conducted to examine the relationship of running kinematics and muscle activation on the modifiable risk factors of HSI on. The overall model (F_2,19_) was statistically significant for both relative eccentric hamstring strength (F = 23.58, *p* < 0.001) and BF_LH_ fascicle length (F = 18.87, *p* < 0.001) (Table 3 and Table 4).

### 3.1. Relative Eccentric Hamstring Strength

Significant correlations were found between relative eccentric hamstring strength and several biomechanical variables (Table 3 and Table 4). Greater eccentric hamstring strength was associated with higher stride frequency, reduced peak anterior pelvic tilt, greater hip flexion and knee extension during the swing phase, and smaller thigh separation angles. Additionally, stronger hamstrings were associated with a lower duty factor, greater knee extension velocity during swing and lower peak BF muscle activity.

### 3.2. Biceps Femoris Fascicle Length

Significant correlations were found between BF_LH_ fascicle length and several biomechanical variables (Table 3 and Table 4). Greater fascicle length was associated with reduced peak anterior pelvic tilt, greater hip flexion and knee extension velocity during the swing phase, along with lower BF muscle activity and lower BF:MH ratio.

## 4. Discussion

In agreement with the hypotheses, spatiotemporal characteristics, running kinematics and hamstring activation patterns were significantly related to both strength and architectural factors. The overall model (F_2,19_) was statistically significant for both relative eccentric hamstring strength (F = 23.58−24.91, *p* < 0.001) and BF_LH_ fascicle length (F = 18.87, *p* < 0.001). Peak anterior pelvic tilt, thigh separation angle, duty factor, peak BF EMG and the activation ratio had negative relationships with relative eccentric hamstring strength (Table 3), whereas stride frequency, peak hip flexion, peak knee extension and peak knee extension velocity were positively correlated with eccentric hamstring strength. Peak anterior pelvic tilt, peak BF EMG and the activation ratio had negative relationships with BF_LH_ fascicle length, whereas peak hip flexion and knee extension velocity were positively associated with BF_LH_ fascicle length. These findings highlight that practitioners need to consider the interrelationship between running kinematics, physical qualities, and muscle architectural factors associated with HSI.

The results of the present study provide novel information regarding the association between spatiotemporal characteristics, eccentric hamstring strength, and BF_LH_ fascicle length. Spatiotemporal characteristics such as stride frequency and duty factor were significantly related to eccentric hamstring strength with stride frequency positively associated with relative eccentric hamstring strength. With every 1 SD increase in stride frequency there was a 1.1–3 SD increase in eccentric hamstring strength. This is consistent with the results of Alt et al. [44] where there was a positive relationship between eccentric hamstring strength and thigh angular velocity in elite sprinters. Greater eccentric hamstring strength was also associated with lower duty factor. This may be explained by stronger athletes possessing greater capacity to develop higher knee flexor torque and limb angular velocities during swing, facilitating greater force application during stance [58,59]. This finding is consistent with prior research highlighting the significant role of the hamstrings in developing horizontal force during acceleration and the observed reductions in horizontal force production following injury [37,60]. These relationships suggest a link between spatiotemporal parameters and physical qualities; however, it remains unclear whether these represent causal or correlational associations. For instance, athletes with greater running economy, neuromuscular coordination or a history of sprint training may naturally demonstrate higher step frequencies and greater eccentric hamstring strength, therefore, representing correlational associations. Conversely it is plausible that greater eccentric hamstring strength may directly contribute to step frequencies through allowing rapid limb repositioning and greater force production during stance.

Spatiotemporal characteristics can be used to classify athletes’ performance; Wild et al. [61] observed that athletes with increased step frequency had a reduced duty factor, which was also associated with increased isomeric hip extensor torque and isomeric hip extensor torque relative to contact time. These findings collectively support the notion that spatiotemporal characteristics are associated with eccentric hamstring strength, particularly higher stride frequency and lower duty factor having positive relationships with eccentric hamstring strength. Alt et al. [44] observed that eccentric hamstring strength had a weak positive association to sprint velocity, while knee extension velocity during the late swing phase had strong positive association with maximal sprint velocity in regional-national level sprinters (100 m times = 10.69–12.66 s). These findings collectively support the notion of a performance–injury trade-off, where if athletes display front-side mechanics, which are positively associated with performance, they must possess high levels of eccentric hamstring strength and BF_LH_ fascicle length. However, as correlation does not equate to causation, further investigation is required to determine if there is a cause or consequence link with running mechanics and the observed architectural and physical factors.

An interesting observation of the present study was that greater anterior pelvic tilt and increased thigh separation angles were significantly associated with reduced relative eccentric hamstring strength, while anterior pelvic tilt was also negatively associated with BFLH fascicle length. These kinematic features are characteristic features of “back-side” running mechanics, which are frequently associated with HSIs [36,62,63,64]. One possible interpretation is that these kinematic patterns may reflect physical and architectural adaptations to individual movement patterns. Specifically, increases in muscle fascicle length are thought to occur when angular excursions or loading demands are high at long muscle lengths [65,66,67]. However, back-side running mechanics typically result in reduced hip and knee flexion angles during late swing, leading to the hamstring musculature operating at shorter muscle lengths. This may result in eccentric force production at reduced lengths and potential result in the development of shorter muscle fascicle lengths.

The apparent discrepancy between the shortened operating lengths observed with back-side mechanics and the theoretical expectation of fascicle lengthening under large angular excursions may be explained by the timing and distribution of load within the muscle–tendon unit. In back-side mechanics, the hamstrings are likely to experience peak loading at relatively smaller hip flexion angles and therefore shorter muscle lengths during the swing phase. Therefore, despite substantial joint excursions the fascicles themselves undergo less strain at long lengths, creating a weaker stimulus for architectural adaptations. In contrast, front-side mechanics are characterised by greater hip flexion and knee extensions angles during late swing, which may promote eccentric loading at longer muscle lengths, creating a greater stimulus for fascicle lengthening and adaptations.

Prior research has demonstrated sprint running alone can induce increases in both BF_LH_ fascicle length and eccentric hamstring strength [68], suggesting the mechanical demands of running can influence muscle architecture. This is in agreement with resent evidence demonstrating differences in hamstring muscle morphology between sprinters and team sport athletes [45,46] and showing differences in sprint running kinematics between team sport and sprint-based athletes [69]. Based on data from these studies, and that of the current investigation, it is possible that individual mechanical patterns adopted may influence architectural and physical adaptations through the tissue specific demands imposed during running. If this is the case, it would challenge conventional injury prevention conditioning strategies which aim to increase both muscle fascicle length and eccentric strength, as these physical and architectural adaptations may not be optimised for the functional demands of back-side running mechanics.

Alternatively, it is plausible that these mechanics may arise because of reduced eccentric strength and shorter fascicles. The observed association between increased duty factor, a surrogate for longer stance durations, and reduced eccentric strength suggests that athletes with lower strength may rely on prolonged ground contact times to generate sufficient propulsive force. This compensatory strategy may naturally increase toe-off distance, reinforcing a back-side running pattern [36]. This inter-relationship between kinematic patterns and factors such as eccentric strength and fascicle length suggests a more complex, bidirectional relationship than previously considered. Specifically, for each 1 SD increase in anterior pelvic tilt, there was a significant reduction in eccentric hamstring strength (Right: β = −0.94, *p* = 0.010; Left: β = −0.99, *p* = 0.003) and BF_LH_ fascicle length (Right: β = −0.68, *p* = 0.038). A similar trend was seen with thigh separation angle, further reinforcing the potential mechanistic role of running kinematics in influencing hamstring muscle architecture. Collectively, these findings highlight the need to consider individual running mechanics when designing HSI prevention programs and support the idea of an interaction between biomechanics and muscle–tendon properties.

Maximising front-side mechanics has theoretical implications on performance [70,71] and may reduce proximal hamstring tissue strain [72]. However, as the hamstring MTU lengthens during the late swing phase through tendon stretch, distal tissue strain may increase [15]. In the present study, kinematics associated with frontside mechanics, such as peak hip flexion and knee extension [70], were positively associated with eccentric hamstring strength and BF_LH_ fascicle length. Bramah and colleagues [36] identified that peak hip flexion could present as a performance-injury paradox, whereby hip flexion may increase limb angular acceleration and resultant vertical ground reaction forces, while also increasing hamstring MTU lengths and the requirement for negative work. Improved strain capacity, through increased eccentric hamstring strength and BF_LH_ fascicle length, may represent a greater force expression through greater muscle cross sectional area. This could enable more force generation and energy storage and return in the elastic component. This would align with the current observations of an association between peak hip flexion, knee extension, and knee extension velocities with eccentric strength and BF_LH_ fascicle length. With these kinematic features the requirement for greater relative eccentric hamstring strength in order to effectively decelerate the momentum of the shank during the late swing phase is likely greater [73,74], especially during the descending limb of the length-tension curve where the ability to apply force at longer muscle length can be attributed with increased BF_LH_ fascicle length [75]. The increased limb velocities likely contribute to the force applied during the stance phase via faster limb positioning for optimal application of force in the early stance phase [59,76]. Effectively transferring force during the stance phase is likely to be influenced by other moderating factors, such as neuromuscular control, technique, and fatigue; however, it remains unknown on the strength of these mediating factors play a significant role on spatiotemporal factors.

Greater relative activation of the BF_LH_ was negatively associated with eccentric hamstring strength, where 1 SD increase in BF_LH_ activation was associated with a decrease in eccentric hamstring strength of between 0.84 and 0.86 × SD. This finding indicates that with decreased eccentric hamstring strength there is an increase requirement for the BF_LH_ to decelerate the shank, which could increase the risk of HSI. This is an important consideration as relative contribution of the medial hamstrings (ST) is typically greater than the contribution of the BF_LH_ during running tasks [9,32]. Our finding of lower BF:ST ratio being associated with higher strength values and longer fascicle lengths may suggest that stronger athletes tend to recruit the medial hamstrings more effectively, which may contribute to greater load sharing between the hamstring musculature; potentially reducing strain on the BF_LH_. In contrast, reduced eccentric hamstring strength could indicate that the increased BF_LH_ activation is required to resist the increasing tensile force in the late swing phase as the medial hamstrings are not sufficiently strong enough to do so.

The identified associations between running mechanics, hamstring strength, and architectural properties suggest meaningful interactions that should be considered when managing individual athletes. These relationships may reflect either: 1, mechanical patterns adopted because of an athlete’s existing physical and architectural features, or 2, physical and architectural adaptations shaped by the tissue specific demands imposed by different running mechanics. If the former is true, practitioners should consider aligning technical development with targeted strength and architectural adaptations, ensuring athletes have the physical capacity to adopt front-side running mechanics while minimising injury risk. Conversely, if the latter is true, it would challenge conventional injury prevention strategies which aim to increase both muscle fascicle length and eccentric strength, as these physical and architectural adaptations may not be optimised for the functional demands of mechanical features such as back-side running mechanics. These findings highlight the importance of individualised conditioning approaches and highlight the need for future interventional research to investigate how running kinematics, strength and architecture respond to either physical quality development or technique-based interventions. Previously published intervention studies have shown to result in positive changes to running kinematics, strength, and architecture [68,77,78,79]; however, the changes to the applied strain (i.e., running kinematics) or strain capacity (i.e., hamstring strength and architecture) of the hamstrings has only observed independently making identifying the primary drivers for adaptation and supporting any causal mechanism difficult to determine.

This study is not without its limitations; the top speed of the treadmill was only 18 km·h^−1^, which by team sport standards is lower than the high-speed running velocities, being potentially categorised into striding within team sports [79]. Secondly, it has been suggested previously that there are differences in running biomechanics observed between treadmill and over-ground running, despite the research being conflicting. Van Hooren et al. [80] identified that spatiotemporal, kinematic, kinetic, muscle activity, and muscle–tendon outcome measures are largely comparable between treadmill and overground running, although several sagittal plane outcome measures differed between treadmill and overground running [80]. Furthermore, hip angle at TO was greater when running on a treadmill when compared to track-based running [80]. The findings could indicate that some of the observations in sagittal plane running kinematics may at the very least be exaggerated when running on a treadmill [80]. The present study is limited by the fact that only select peak kinematic features were observed, which only occur for a fraction of the time within the stride. Therefore, it is recommended that waveform analysis should be performed to further investigate the difference and the magnitude of such differences across the gait cycle.

## 5. Conclusions

To the authors’ knowledge, this is the first study to investigate whether spatiotemporal, pelvic, and lower limb kinematics and hamstring muscle activation are associated with hamstring physical (eccentric strength) and architectural (BF_LH_ fascicle length) properties relevant to HSI risk. The findings indicate that the spatiotemporal characteristics, running kinematics, and peak hamstring activation are significantly related to both relative eccentric hamstring strength and BF_LH_ fascicle length. Kinematic features commonly associated with back-side mechanics, such as anterior pelvic tilt and thigh separation angle, were negatively associated with eccentric hamstring strength, with anterior pelvic tilt also showing a negative association with BF_LH_ fascicle length. In contrast, kinematic features associated with frontside mechanics (i.e., peak hip flexion and peak knee extension velocity) were positively associated with eccentric hamstring strength and BF_LH_ fascicle length. These findings collectively highlight the complex interrelationship between running mechanics and hamstring muscle properties, with the potential of both a “cause”, whereby muscle strength and architectural properties represent adaptations to mechanics, and a “consequence”, where the mechanical patterns are adopted based on the existing physical and architectural features. Given the performance-injury trade-off between front-side versus back-side mechanics, practitioners should consider aligning technique development with targeted strength and architectural adaptations to improve performance while mitigating injury risk.

## Figures and Tables

**Table 1:** Kinematic running metric identified using 3D motion data.

Metric	Description
Stride Frequency (Hz)	Average steps per minute
Stride Length (m)	Average step length based on running velocity and step frequency
Peak Anterior Pelvic tile (°)	Maximal anterior angle on the pelvis in the sagittal plane, 0° = neutral
Peak Hip Extension (°)	Maximal hip extension, 0° = neutral
Peak Hip Flexion (°)	Maximal hip flexion, 0° = neutral
Peak Knee Extension (°)	Maximal knee extension, 180° = full extension
Peak Knee Flexion (°)	Maximal knee flexion, 180° = full extension
Thigh Separation Angle at touch down (°)	Difference hip flexion angle of lead leg and hip extension angle of the trailing leg
Duty Factor (%)	Relative contact time to total stride time (swing time + ground contact time)
Knee Extension Velocity (°/s)	Knee extension velocity of the leading leg during the late swing phase

**Table 2:** Descriptive data (Mean ± Standard deviation [SD]) and absolute and relative reliability for modifiable risk factors and running kinematics and hamstring muscle activation.

	Mean ± SD		ICC (95% CI)	CV% (95% CI)
	Left	Right		
BF_LH_ fascicle length (cm)	9.94 ± 1.73	10.10 ± 1.76	0.881 (0.813–0.952)	1.88 (0.77–2.99)
Relative eccentric hamstring strength (Nm/Kg)	1.57 ± 0.31	1.51 ± 28	0.965 (0.904–0.989)	1.39 (0.36–2.42)
Stride frequency (Hz)	90.10 ± 5.32	93.99 ± 5.93	0.813 (0.743–0.908)	4.68 (2.89–6.43)
Stride length (m)	1.46 ± 0.10	1.43 ± 0.06	0.842 (0.789–0.895)	4.21 (2.91–5.48)
Peak anterior pelvic tilt (°)	-6.49 ± 2.29	−6.42 ± 2.71	0.828 (0.756–0.831)	7.28 (5.12–9.54)
Peak hip extension (°)	−10.28 ± 6.68	−9.23 ± 5.49	0.902 (0.877–0.954)	3.89 (3.72–4.06)
Peak hip flexion (°)	55.55 ± 5.29	54.32 ± 5.83	0.869 (0.810–0.900)	7.08 (6.91–7.25)
Peak knee extension (°)	114.59 ± 5.95	110.50 ± 5.51	0.819 (0.726–0.871)	7.86 (7.69–8.03)
Peak knee flexion (°)	13.59 ± 2.30	11.36 ± 2.42	0.869 (0.803–0.901)	2.74 (2.57–2.91)
Thigh separation angle (°)	65.83 ± 5.18	63.55 ± 6.83	0.793 (0.713–0.878)	6.72 (3.69–9.95)
Duty factor (%)	15.62 ± 2.48	14.68 ± 2.50	0.919 (0.879–0.946)	4.46 (3.93–4.99)
Knee extension velocity (°/s)	919.45 ± 66.39	911.55 ± 53.19	0.912 (0.870–0.960)	5.01 (3.46–6.40)
Peak BF EMG (%)	74.40 ± 16.51	67.51 ± 27.58	0.809 (0.773–0.855)	5.88 (5.52–6.02)
Peak MH EMG (%)	69.48 ± 23.51	61.41 ± 20.80	0.789 (0.736–0.841)	6.80 (6.29–7.33)
Activation ratio (BF:MH) (%)	1.14 ± 0.35	1.17 ± 0.44	0.819 (0.772–0.861)	7.16 (6.81–7.45)

BF_LH_ = Bicep femoris long head, MH = Medial hamstrings.

**Table 3:** Multiple linear regression for running kinematics and the modifiable risk factors of hamstring strain injuries.

		Relative Eccentric Hamstring Strength			Bicep Femoris Fascicle Length		
Metric	Limb	*β* (95% CI)	*t*	*p*	*β* (95% CI)	*t*	*p*
Stride frequency (Hz)	Right	1.34 (0.78–1.83)	3.32	0.003 *	0.57 (−0.30–1.13)	0.56	0.081
	Left	1.17 (0.62–1.54)	2.76	0.012 *	0.57 (−0.30–1.13)	0.53	0.078
Stride length (m)	Right	0.31 (−1.28–1.63)	0.52	0.319	0.31 (−1.03–1.58)	0.63	0.534
	Left	0.27 (−1.05–1.36)	0.59	0.413	0.36 (−1.29–1.63)	1.32	0.202
Peak anterior pelvic tilt (°)	Right	−0.94 (−1.55–0.31)	−2.45	0.010 *	−0.68 (−1.12–0.25)	0.90	0.038 *
	Left	−0.99 (−1.65–0.32)	−3.41	0.003 *	−0.69 (−1.02–0.29)	0.89	0.038 *
Peak hip extension (°)	Right	0.33 (−1.44–2.06)	0.95	0.094	0.38 (−1.41–1.86)	0.80	0.078
	Left	0.32 (−1.37–1.86)	0.97	0.084	0.48 (−1.13–2.06)	1.15	0.065
Peak hip flexion (°)	Right	0.64 (0.15–1.06)	1.45	0.024 *	0.58 (0.12–1.03)	1.61	0.038 *
	Left	0.58 (0.11–0.93)	1.72	0.014*	0.60 (0.16–1.04)	1.68	0.023 *
Peak knee extension (°)	Right	0.71 (0.19–1.26)	2.53	0.022 *	0.52 (−0.22–1.23)	1.37	0.109
	Left	0.76 (0.22–1.29)	2.31	0.033 *	0.53 (−0.18–1.24)	1.83	0.084
Peak knee flexion (°)	Right	0.30 (−1.07–1.66)	0.71	0.264	0.21 (−1.05–1.46)	0.70	0.371
	Left	0.31 (−1.05–1.67)	0.73	0.259	0.21(−1.10–1.62)	0.59	0.328
Thigh separation angle (°)	Right	−0.73 (−1.28–0.19)	−0.97	0.023 *	0.86 (−0.22–1.76)	1.73	0.099
	Left	−0.74 (−1.28–0.20)	−0.95	0.028 *	0.70 (−0.12–1.64)	1.50	0.123
Duty factor (%)	Right	−0.62 (−1.05–0.22)	−3.65	0.002 *	0.80 (−0.04–1.66)	2.04	0.055
	Left	−0.59 (−1.00–0.19)	−2.38	0.008 *	0.56 (−0.38–1.46)	1.21	0.240
Knee extension velocity (°/s)	Right	0.53 (0.04–1.01)	3.14	0.005 *	0.99 (0.06–1.76)	2.18	0.042 *
	Left	0.54 (0.05–1.01)	2.53	0.020 *	0.69 (0.02–1.41)	1.50	0.028 *

* = denotes a significant prediction.

**Table 4:** Multiple linear regression for hamstring activation and the modifiable risk factors of hamstring strain injuries.

		Relative Eccentric Hamstring Strength			Bicep Femoris Fascicle Length		
Metric	Limb	*β* (95% CI)	*t*	*p*	*β* (95% CI)	*t*	*p*
Peak BF EMG (%)	Right	−0.85 (−1.38–0.40)	−2.37	0.018 *	−0.57 (−1.06–0.11)	1.64	0.029 *
	Left	−0.86 (−1.35–0.43)	−2.32	0.031 *	−0.51 (−1.02–0.08)	1.12	0.027 *
Peak MH EMG (%)	Right	−0.10 (−0.95–0.86)	−0.27	0.499	0.26 (−0.85–1.04)	0.44	0.665
	Left	−0.11 (−0.94–0.85)	−0.25	0.608	0.39 (−0.93–1.37)	1.03	0.317
Activation ratio (BF:MH) (Au)	Right	−0.44 (−1.08–0.23)	−1.45	0.066	−0.35 (−1.03–0.02)	1.28	0.048 *
	Left	−0.46 (−1.10–0.19)	−1.56	0.053	−0.40 (−0.83–0.01)	1.77	0.045 *

* = denotes a significant prediction.

## Data Availability

The raw data supporting the conclusions of this article will be made available by the authors on request.

## Abbreviations

The following abbreviations are used in this manuscript:

HSI	Hamstring strain injury
BFLH	Bicep femoris long head
MH	Medial hamstrings
MTU	Muscle tendon unit
ST	Semitendinosus
TD	Touch down
TO	Take off
